# Daytime pattern of post-exercise protein intake affects whole-body protein turnover in resistance-trained males

**DOI:** 10.1186/1743-7075-9-91

**Published:** 2012-10-16

**Authors:** Daniel R Moore, Jose Areta, Vernon G Coffey, Trent Stellingwerff, Stuart M Phillips, Louise M Burke, Marilyn Cléroux, Jean-Philippe Godin, John A Hawley

**Affiliations:** 1 Nestlé Research Centre, Nestec Ltd, Lausanne, Switzerland; 2 Human Health and Nutritional Sciences, University of Guelph, 50 Stone Road East, Guelph, Ontario, N1G 2W1, Canada; 3 Exercise and Nutrition Research Group, School of Medical Sciences, RMIT University, Melbourne, VIC, Australia; 4 Canadian Sport Centre - Pacific, Victoria, British Columbia, Canada; 5 Department of Kinesiology, McMaster University, Hamilton, ON, Canada; 6 Sports Nutrition, Australian Institute of Sport, Belconnen, ACT, Australia

**Keywords:** Protein intake, Protein metabolism, Exercise training, Hypertrophy, Lean body mass

## Abstract

**Background:**

The pattern of protein intake following exercise may impact whole-body protein turnover and net protein retention. We determined the effects of different protein feeding strategies on protein metabolism in resistance-trained young men.

**Methods:**

Participants were randomly assigned to ingest either 80g of whey protein as 8x10g every 1.5h (PULSE; n=8), 4x20g every 3h (intermediate, INT; n=7), or 2x40g every 6h (BOLUS; n=8) after an acute bout of bilateral knee extension exercise (4x10 repetitions at 80% maximal strength). Whole-body protein turnover (Q), synthesis (S), breakdown (B), and net balance (NB) were measured throughout 12h of recovery by a bolus ingestion of [^15^N]glycine with urinary [^15^N]ammonia enrichment as the collected end-product.

**Results:**

PULSE Q rates were greater than BOLUS (~19%, P<0.05) with a trend towards being greater than INT (~9%, P=0.08). Rates of S were 32% and 19% greater and rates of B were 51% and 57% greater for PULSE as compared to INT and BOLUS, respectively (P<0.05), with no difference between INT and BOLUS. There were no statistical differences in NB between groups (P=0.23); however, magnitude-based inferential statistics revealed likely small (mean effect±90%CI; 0.59±0.87) and moderate (0.80±0.91) increases in NB for PULSE and INT compared to BOLUS and possible small increase (0.42±1.00) for INT vs. PULSE.

**Conclusion:**

We conclude that the pattern of ingested protein, and not only the total daily amount, can impact whole-body protein metabolism. Individuals aiming to maximize NB would likely benefit from repeated ingestion of moderate amounts of protein (~20g) at regular intervals (~3h) throughout the day.

## Background

Recommendations for dietary protein intake for healthy adults currently provide guidelines according to the total daily quantity of protein intake [1,2], while new guidelines for athletes also note the importance of protein intake immediately after a resistance training session [3]. However, beyond total daily protein intake, a more balanced pattern of protein intake throughout the day can significantly improve net nitrogen retention of isonitrogenous diets in children and adults [4,5], although this finding is not universal [6,7]. Moreover, tendencies toward a greater nitrogen balance and a lower whole-body protein breakdown have been observed in young women ingesting their daily protein intake in 4 balanced meals as compared to focussed in a single meal [8]. Collectively, these studies [4,5,8] highlight the need to further investigate the impact different feeding patterns may have on protein kinetics to elucidate their physiological relevance for individuals aiming to maintain or increase lean body mass.

Given the known effect of dietary protein on the enhancement of muscle and whole-body protein synthesis [9] and the apparent advantage to balanced protein ingestion for whole-body nitrogen retention [5], we provided trained young men with isonitrogenous amounts of high quality protein that differed only in the pattern of ingestion to determine its effect on whole-body protein metabolism over a prolonged postprandial period. Knowing the dose–response relationship for optimally enhancing rested and post-exercise muscle protein synthesis [10,11], the protein feeding patterns were designed to provide repeated feedings of surfeit (40g), optimal (20g), or insufficient (10g) per meal protein intakes [10,11]. Based on previous findings [10,11] and that skeletal muscle is the major storage reservoir for body amino acids, we hypothesized that whole-body net protein balance would be greatest with repeated feedings of 20g of protein. The period of investigation was in the 12h period immediately following a bout of resistance exercise to standardise and take advantage of the accentuated protein synthesis in the exercised muscle over this period [12].

## Methods

Twenty-four male subjects participating in regular high-intensity resistance training (4–6 times per week) were recruited for this study and provided their written, informed consent. The study conformed to the standards set by the Declaration of Helsinki, and carried the approval of the Human Research Ethics Committee of RMIT University and the Australian Institute of Sport.

After reporting to the laboratory to undergo a whole-body DEXA scan [13] and to determine one-repetition maximum (1-RM) bilateral knee extensor strength, subjects were provided with a standardized diet for the 72h prior to the trial that provided an energy availability of 45 kcal/kg fat-free mass with a macronutrient contribution 1.5 g protein/kg/d and 4 g carbohydrate/kg/d , respectively. Subjects were instructed to refrain from training and other vigorous physical activity during the 72h period.

Subjects reported to the laboratory after a 10-h overnight fast and provided a spot urine sample to determine baseline enrichment (see below). Following two warm up sets (5 repetitions at 60% and 70% 1-RM), subjects completed an acute bilateral leg extension exercise session (4x10 sets at 80% 1-RM with 3 min recovery between sets). Subjects were randomly allocated to receive a total of 80g of whey protein isolate over 12h in one of three different ingestion patterns: pulsed feeding (PULSE), 8x10g every 1.5h; intermediate feeding (INT), 4x20g every 3h ; or bolus feeding (BOLUS), 2x40g every 6h. The BOLUS group was designed to simulate the ingestion of 2 large meals of the three meals one might consume in a given day. The first beverage of each group was consumed immediately after exercise and included 200mg of [^15^N]glycine. Subjects rested comfortably in the laboratory and collected all urine over the complete 12-h period, which was pooled and stored at −80°C until further analysis.

Urinary ammonia was isolated by cation exchange resin with the ^15^N]enrichment determined by isotope ratio mass spectrometry [14]. Whole-body protein turnover (Q) was calculated using the ^15^N]ammonia end-product method as described previously [15], which provides consistent rates over 12h [16]. Concentrations of urinary urea and creatinine, the major nitrogen containing metabolites in urine, were measured by automated analyser at the Laboratoire Central de Chimie Clinique (Centre Hospitalier Universitaire Vaudois, Lausanne, Switzerland). Whole-body protein synthesis (S) and protein breakdown (B) were calculated as previously described [14] with estimated fecal and miscellaneous nitrogen losses of 0.95g/12h and 0.69g/12h, respectively, which were based on half the daily excretion previously measured in strength athletes consuming a moderate protein diet [14]. Whole-body net protein balance (NB) was calculated as: S minus B. Data were expressed normalized to both whole body mass (BM) and fat- and bone-free (i.e., lean) body mass (LBM).

Data were analyzed using a one-way repeated measure analysis of variance (ANOVA) with Student Newman Keuls post-hoc analysis (Sigmastat V3.11). In the event of non-normal distribution, data were log-transformed prior to analysis. Statistical significance was established at P<0.05 and all data are expressed as mean ± standard deviation. It has been demonstrated previously that the ^15^N]glycine-measured increase in NB is able to reasonably predict the training-induced increase in lean body mass in young men [17]. To account for possible subtle differences in the most physiologically meaningful variable of NB, mean effect sizes and 90% confidence intervals (CI) were also calculated [18] to allow for probabilistic magnitude-based inferences between groups [18,19]. Quantitative chances of benefit and harm were assessed according to previously published cut-points using the smallest standardized change in the mean, as described in detail elsewhere [18,19].

## Findings

Due to a technical limitation, one subject in the INT group did not receive the appropriate feeding regimen and was removed from analysis. There was no difference in total (82.0±6.4, 77.5±7.9, and 83.6±10.5kg) or lean body mass (66.5±5.3, 64.0±5.8, and 66.2±5.4kg) for the participants in the PULSE, INT, and BOLUS groups, respectively. The 80g of protein provided the subjects with 65±5%, 69±8%, and 65±8% of the subject’s mean daily protein intake (at 1.5.g/kg/d) of the controlled diet in the PULSE, INT and BOLUS groups, respectively (P=0.39).

**Figure 1:**
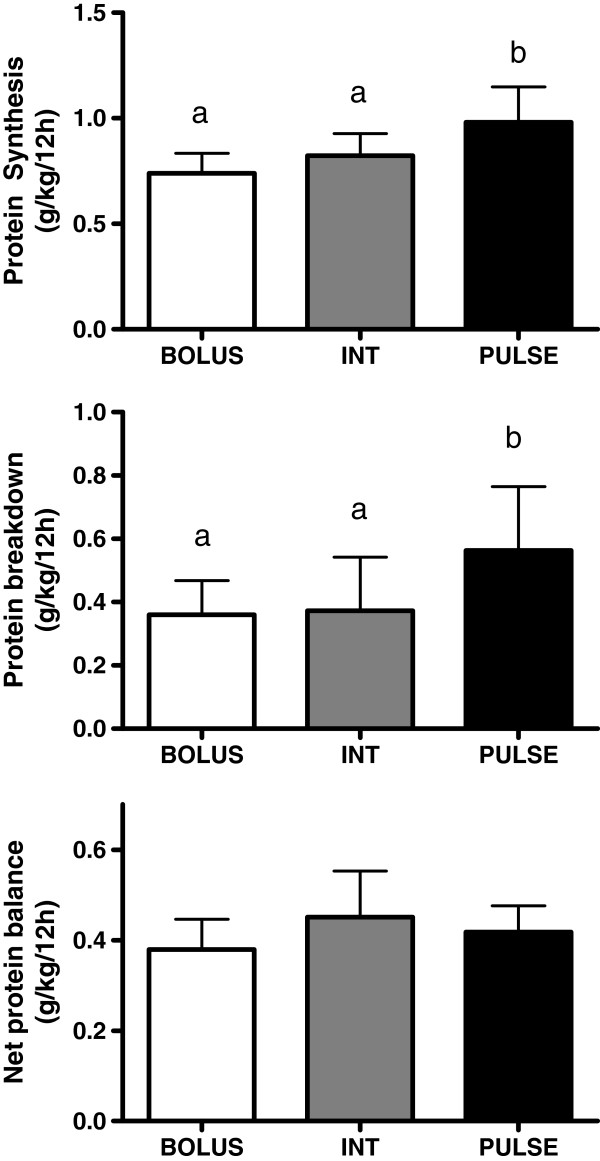
**Whole body protein synthesis (top), protein breakdown (middle) and protein balance (bottom) expressed per kg body mass over a 12 h postprandial period.** Values are mean ±SD. Means with different letters are significantly different (P<0.05) according to one-way ANOVA with Student-Newman Keuls post-hoc.

When expressed relative to BM, whole-body nitrogen turnover (Q) was greater with PULSE as compared to BOLUS (0.25±0.03 vs 0.21±0.02g N/kg BW/12h; P<0.05) there was a trend towards a difference between PULSE and INT (0.23±0.1; P=0.08). PULSE resulted in a ~19 and ~32% greater (P<0.05) whole-body protein synthesis (S) compared to INT and BOLUS, respectively (Figure 1A). There was no difference between INT and BOLUS for S (P=0.17). Similarly, whole-body protein breakdown (B) was ~51 and ~57% higher (P<0.05) with PULSE as compared to INT and BOLUS, respectively. However, there was no difference between INT and BOLUS for B (P=0.88). There were no differences (P=0.23) in NB between any conditions using an ANOVA. Analysis of the same data using probabilistic magnitude-based inferences revealed likely moderate and small positive effects for INT and PULSE, respectively, compared to BOLUS for NB (Table 1). Moreover, there was a possible small increase in NB for INT as compared to PULSE. When the data were expressed relative to lean body mass there was ~21-25% increase in all rates (Q, S, B) and NB due to the normalization to a lower total mass (i.e. lean vs whole body) but with similar differences and effects between groups (data not shown).

**Table 1:** Effect of feeding pattern on whole body net protein balance

**Comparison**	**Mean effect±CI**	**P**-**value**	**Qualitative inference**
PULSE - BOLUS	0.59±0.87	0.25	Small increase likely
INT - BOLUS	0.80±0.91	0.15	Moderate increase likely
INT - PULSE	0.42±1.00	0.46	Small increase possible

Effect size thresholds: <0.2, trivial; <0.6, small; <1.2, moderate; <2.0, large.

## Discussion

We report here for the first time that manipulation of the pattern of intake of an identical amount of high quality protein over a 12-h post-prandial period following a bout of resistance exercise altered whole-body protein metabolism in trained young men. Our observation of larger protein feedings (i.e. ≥20g; INT and BOLUS) eliciting lower whole-body protein turnover than smaller (i.e. 10g; PULSE) repeated feedings may be related in part to a nutrient partitioning of dietary amino acids away from metabolism within non-muscle tissues with higher turnover rates (e.g. splanchnic region) towards skeletal muscle, which turns over relatively slowly [20]. We have proposed such a possibility previously in trained populations who presented lower rates of whole body protein turnover post-training but with concurrent enhancements in net balance and lean mass gains [14,21]. However, in light of the relative dearth of information on the physiological impact of different feeding patterns, substantiation of this hypothesis would require future studies that manipulate dietary ingestion patterns with concurrent measures of whole body and tissue-specific (e.g. muscle) protein metabolism.

A limitation of whole-body tracer methodologies is the inability to delineate tissue-specific changes in protein metabolism. However, the advantage is the ability to measure metabolism of the whole system to determine its net anabolic or catabolic state. The positive net balance in treatment groups is consistent with the anabolic effects of feeding and protein ingestion that would subsequently result in protein synthesis and protein deposition within both rested and exercised lean tissues [11]. Regardless, despite this anabolic environment, subtle differences in feeding pattern on whole-body protein balance were evident in that repeated feedings of 10 and 20g of protein resulted in a likely greater protein balance compared to larger 40g feedings (Table 1). The physiological basis for these differences may be related in part to lower amino acid oxidation that would occur with the smaller (i.e. ≤20g) repeated feedings [11]. In contrast, the possibly greater protein balance with the 20g as compared to the 10g feedings may be related to a more efficient stimulation of protein synthesis in both rested and exercised skeletal muscle and hence deposition of dietary amino acids within that tissue [11,22,23]. In light of our previous observations of a reasonable agreement between tracer-derived increases in whole-body net protein balance (measured by oral ^15^N]glycine) and training-induced increases in lean body mass [14], the present data could suggest that individuals wishing to enhance or maintain lean body mass could obtain a benefit from the repeated ingestion of moderate amounts of dietary protein at regular intervals throughout the day. This concept has been raised previously for athletes [3], may have preliminary support in general populations [24], and represents, we propose, a fruitful area of further study.

## Conclusions

In conclusion, despite equivalent total protein intake, whole-body protein synthesis and breakdown are greatest when small (i.e. 10g) as compared to larger (i.e. ≥20g) protein feedings are consumed at regular intervals during a 12h postprandial period after a bout of exercise. However, whole-body protein balance tended to be greatest with moderate 20g feedings every 3h, which may have implications for individuals aiming to enhance whole-body anabolism including lean body mass accrual with training. Collectively, our data highlight that the acute pattern, and not only the total amount, of ingested protein should be considered when determining feeding strategies to alter whole-body protein metabolism.

## Abbreviations

PULSE: Protein ingestion pattern of 10g of protein at 1.5h intervals for 12h (i.e. 8 feedings); INT: Protein ingestion pattern of 20g of protein at 3h intervals for 12h (i.e. 4 feedings); BOLUS: Protein ingestion patter of 40g of protein at 6h intervals for 12h (i.e. 2 feedings); Q: Whole body nitrogen turnover; S: Whole body protein synthesis; B: Whole body protein breakdown; NB: Whole body net protein balance; LBM: Lean body mass; BM: Body mass.

## Competing interests

Some of the authors (MC, and JPG) are or were (DRM, TS) employees of Nestec Ltd., which is a subsidiary of Nestlé Ltd. and provides professional assistance, research, and consulting services for food, dietary, dietetic, and pharmaceutical products of interest to Nestlé Ltd. The authors declare no financial conflicts of interest.

## Authors’ contributions

DRM, VGC, TS, SMP, LMB, and JAH designed the study and obtained the grant. JLA, VGC, LMB, and JAH carried out the study. DRM, JLA, VGC, MC, and JPG conducted laboratory analysis. DRM, SMP, and JAH performed statistical analysis and wrote the manuscript. All authors read and approved the final manuscript.
